# Effects of different wheat bran fermentation sources on growth performance, nutrient digestibility, serum antioxidant capacity and fecal microbiota in growing pigs

**DOI:** 10.3389/fvets.2023.1289010

**Published:** 2023-11-16

**Authors:** Heng Liu, Xiaojie Ren, Yang Li, Qingjie Cao, Lijie Yang, Shuzhen Jiang, Jiawei Fu, Jie Gao, Lei Yan, Junxun Li, Weiren Yang

**Affiliations:** ^1^Key Laboratory of Efficient Utilization of Non-grain Feed Resources (Co-construction by Ministry and Province), Ministry of Agriculture and Rural Affairs, Shandong Provincial Key Laboratory of Animal Biotechnology and Disease Control and Prevention, Department of Animal Science and Veterinary Medicine, Shandong Agricultural University, Tai’an, China; ^2^Shandong Taishan Shengliyuan Group Co., Ltd., Tai’an, China; ^3^Chambroad Holding Group, Binzhou, China; ^4^College of Biology and Brewing Engineering, Mount Taishan College, Tai’an, China; ^5^Shandong New Hope Liuhe Group Co., Ltd., Qingdao, China

**Keywords:** fermented wheat bran, growing pig, growth performance, antioxidant capacity, fecal microflora

## Abstract

The present study aimed to evaluate the application of different wheat bran fermentation sources in growing pigs. A total of 320 pigs (43 ± 0.21 kg), were randomly allocated to 5 groups in a 21-d trial. The control group was fed a basal diet (CON) containing raw wheat bran, and the other four treatments were fed the diets in which the raw wheat bran in the basal diet was substituted with *Aspergillus niger* (WBA), *Bacillus licheniformis* (WBB), *Candida utilis* (WBC), and *Lactobacillus plantarum* (WBL) fermented wheat bran, respectively. The results showed that compared to the CON group, the crude fiber and pH values were decreased (*p* < 0.05), while the gross energy (GE), crude protein (CP), and lactic acid values were increased (*p* < 0.05) in all the wheat bran fermented by different strains. Compared with other treatments, feeding *B. licheniformis* fermented wheat bran had higher final weight, average daily gain, as well as lower feed-to-gain ratio. Compared with CON group, pigs fed with fermented wheat bran diets had higher dry matter, CP, and GE availability, serum total protein, albumin and superoxide dismutase levels, and fecal *Lactobacillus* counts, as well as lower malondialdehyde level and fecal *Escherichia coli* count. Collectively, our findings suggested that feeding fermented wheat bran, especially *B. licheniformis* fermented wheat bran, showed beneficial effects on the growth performance, nutrient digestibility, serum antioxidant capacity, and the gut microbiota structure of growing pigs.

## Introduction

With the steady growth of animal production, feed price is increased (1, 2). Correspondingly, fermentation is recognized as a very effective approach to improve the nutrition and digestion of unconventional feed sources (3–5). Numerous studies have illustrated that the fermented feed improved growth performance, antioxidant capacity, and intestine health of pigs (6–8).

Wheat bran, a byproduct derived from flour processing, is a readily accessible material with an annual global yield of 100–150 million tons (9). Due to its high contents of energy value, crude protein (CP), and trace elements, wheat bran is extensively utilized as animal feed raw materials (10). In feedstuff production, wheat bran processing primarily involves mechanical and chemical methods (11–13). Kraler et al. (14) reported that wheat bran fermented by *Lactobacillus paracasei* and *Lactobacillus plantarum* significantly increased the apparent digestibilities of dry matter (DM), organic matter (OM), ether extract (EE), and gross energy (GE) in growing pigs. Additionally, fermentation could effectively reduce the content of insoluble dietary fiber in rice bran and corn bran (15, 16), and the enzymes produced during fermentation can enhance the absorption of minerals (17). The *Aspergillus niger*, *Bacillus licheniformis*, *Candida utilis* and *L. plantarum* are the four strains used most widely in feed fermentation. Previous studies have demonstrated that the anti-nutritional factors presenting in soybean and sorghum, specifically glycinin, β-conglycinin and tannins, can be effectively degraded by fermentation with *A. niger* or *L. plantarum* (18, 19). Ahmed et al. (20) found that fermented feed by *B. licheniformis* significantly increased growth performance of broilers. The *C. utilis* fermented rapeseed meal improved the intestinal morphology of broilers (21). However, there is little literature available on the comparative study of wheat bran fermented by *A. niger*, *B. licheniformis*, *C. utilis*, and *L. plantarum* on fermentation parameters and their application on growing pigs.

Therefore, the experiment was conducted to investigate the effects of wheat bran fermented by different strains (*A. niger*, *B. licheniformis*, *C. utilis*, and *L. plantarum*) on growth performance, nutrient digestibility, serum antioxidant capacity, and fecal microbiota of growing pigs, and select the optimal strain for wheat bran fermentation, helping to select the most effective fermentative source to improve the feed utilization.

## Materials and methods

### Preparation of fermented wheat bran

The starter culture of *A. niger* (CICC 2041), *B. licheniformis* (CICC 21886), *C. utilis* (CICC 31430), and *L. plantarum* (CICC 6076) used in the test were obtained from Shandong Taishan Shengliyuan Group Co., Ltd. (Tai’an, China), which obtained according to optimized cultivation methods (Supplementary Table S1). Before fermentation, the moisture content of wheat bran was adjusted to 45%. Then the WBA, WBB, WBC, and WBL were fermented according to the conditions shown in Supplementary Table S2. The WBA, WBB, WBC, and WBL products were all obtained by drying at 45°C for approximately 48 to 72 h. Samples of fermented wheat bran were collected to determine their GE, CP, EE, and crude fiber (CF) contents according to the methods described by the Association of Official Analytical Chemists (AOAC) (22). To determine pH, a sample of 5 g fermented wheat bran was dissolved in 50 mL distilled water. The pH value of the supernatant was measured with pH meter (Shanghai Russell Technology Co., Ltd., Shanghai, China) after centrifugation at 4000 *× g* for 5 min. The lactic acid content was determined with a high-performance liquid chromatography autoanalyzer (Waters PICO TAG amino acid autoanalyzer; Millipore, MA, USA) according to standard procedures.

### Experimental design and management

Three hundred and twenty (Duroc × Landrace × Yorkshire) pigs (43 ± 0.21 kg) were randomly divided into 5 treatments with 8 replicates per group and 8 pigs per replicate. Control group was fed a basal diet (CON), and other 4 treatments were fed the diets in which wheat bran of the basal diet were replaced with 10% *A. niger* (WBA), *B. licheniformis* (WBB), *C. utilis* (WBC), and *L. plantarum* (WBL) fermented wheat bran (air-dry basis), respectively. The basal diet (Table 1) was formulated with reference to National Research Council (NRC, 2012). Pigs were housed in a temperature-and humidity-controlled room (26–28°C, 55–60% RH), and were fed three times daily at 0800, 1400, and 1800 h. During the period of the experiment, all pigs had free access to feed and water. Daily feed intake per replicate was recorded, and the pigs were weighted individually on the morning of day 21 of the experiment before breakfast.

**Table 1:** Ingredients and nutrient levels of the basal diet (air-dry basis) %.

Ingredients	Content	Nutrients	Values^2^
Corn	56.57	Metabolizable energy, MJ/kg	13.78
Soybean meal, 44.1% CP	20.65	Crude protein	16.33
Wheat bran	17.00	Calcium	0.56
Soybean oil	2.79	STTD phosphorus	0.24
Limestone, pulverized	1.20	Lysine	0.97
Calcium hydrophosphate	0.17	Methionine	0.32
Sodium chloride	0.20	Threonine	0.72
L-Lysine, 76.8%	0.20	Tryptophan	0.21
DL-methionine, 98.5%	0.07		
L-threonine, 98.0%	0.10		
Choline	0.05		
Premix^1^	1.00		
Total	100.00		

^1^The premix provided the following per kilogram of diets: vitamin A 1400 IU, vitamin B_1_ 1 mg, vitamin B_2_ 2 mg, vitamin B_5_ 7.5 mg, vitamin B_6_ 8.8 mg, vitamin B_12_ 0.008 mg, vitamin D_3_ 160 IU, vitamin E 11 IU, vitamin K_3_ 0.5 mg, biotin 0.05 mg, folic acid 0.3 mg, Fe (FeSO_4_·H_2_O) 60 mg, Cu (CuSO_4_·5H_2_O) 4 mg, Mn (MnSO_4_·H_2_O) 25 mg, Zn (ZnSO_4_·H_2_O) 60 mg, I (KIO_3_) 0.14 mg, Se (Na_2_SeO_3_) 0.25 mg. ^2^Crude protein, calcium and STTD phosphorus were analyzed values, while the other nutrient levels were calculated values.

### Apparent nutrient digestibility

Apparent nutrient digestibility was conducted using acid insoluble ash (AIA) indicator method. Fresh feces excreted by each replicate were collected daily for four consecutive days starting on day 17 of the experiment. Daily collected feces were weighed and mixed, and 10 mL of 10% sulfuric acid per 100 g of the feces samples were added to avoid evaporation of nitrogen in the form of ammonia. Samples were stored temporarily at −20°C. All fecal samples per replicate collected consecutively during the 4 days were mixed evenly, and the nutrient contents of fecal and feed samples including AIA, GE, DM, OM, EE, and CP were analyzed according to AOAC (2012) (22), respectively. The apparent digestibilities of all parameters were calculated as indicated below:


$$\begin{array}{l} \mathrm{Apparent}\;\mathrm{nutrient}\;\mathrm{digestibility}\;\left(\%\right)=\\ 100-100\times \left[\left(AIA-1\times F-2/AIA-2\times F-1\right)\right] \end{array}$$

where AIA-1 was the AIA content of the diets, AIA-2 was the AIA content of the fecal, F-1 was the nutrient content of the diets, and F-2 was the nutrient content of the fecal.

### Sampling procedure

Blood samples were taken from the jugular vein of 40 pigs (one pig per replicate) after being fasted for 12 h in the morning of d 21. About 5 mL of blood samples for hematological tests were collected into the routine blood tubes containing the anticoagulant (EDTA), and the other 10 mL were collected into another vacuum blood collection tube without anticoagulant for biochemical analyses. Serum samples were obtained after centrifugation at 3000 × *g* for 15 min and stored in 1.5 mL Eppendorf tubes at −20°C until analysis. Meanwhile, fresh fecal samples were collected from the 40 pigs by rectal stimulation, and then stored at −20°C until *Escherichia coli* and *Lactobacillus* analysis.

### Serum biochemical parameters analysis

Serum biochemical parameters including alkaline phosphatase (ALP), aspartate amino transferase (AST), alanine aminotransferase (ALT), total protein (TP), albumin (ALB), triglyceride (TG), and total cholesterol (TCHO) were determined with commercial kits (Nanjing Jiancheng Bioengineering Institute, Nanjing, China) using an automatic clinical chemistry analyzer (Roche, Cobus-MiraPlus, Roche Diagnostic System Inc., United States).

### Haemato-immunological blood parameters analysis

The white blood cell count (WBC), lymphocyte ratio (LYN), red blood cell count (RBC), hemoglobin (HGB), mean corpuscular volume (MCV), mean corpuscular hemoglobin (MCH), and mean corpuscular hemoglobin concentration (MCHC) were detected using an Sysmex KX-21 Automated Hematological Analyzer (Abacus Junior Vet, Diatron, Vienna, Austria) with specific software.

### Determination of antioxidant index

The superoxide dismutase (SOD) activity and malondialdehyde (MDA) level of serum were determined by assay kits (Nanjing Jiancheng Biotechnology Institute, China) according to the manufacturer’s instructions.

### Microbiota determination

The frozen fecal samples were incubated at 4°C for 10 h before enumeration. The *E. coli* and *Lactobacillus* were isolated by plating serial tenfold dilution (in sterile physiological saline) onto Luria Broth (LB) agar plates and MacConkey agar plates, respectively. All agar plates were incubated at 37°C for 36 h. The LB agar plates were aerobic incubation, while the MacConkey agar plates was incubated under the anaerobic conditions. Then the *E. coli* and *Lactobacillus* were counted according to the methods of Ganzle et al. (23) and Liu et al. (24).

### Statistical analysis

The data were statistically analyzed using one-way ANOVA of SAS 9.2 (Inst. Inc., Cary, NC). Variations among the 5 treatments were compared with each other using Duncan’s multiple comparisons. The mean and total standard error of the means (SEM) were used to present the results, and the differences between treatments were considered significant when *p* < 0.05.

## Results

### Fermentation parameters and nutrient content of wheat bran

**Figure 1:**
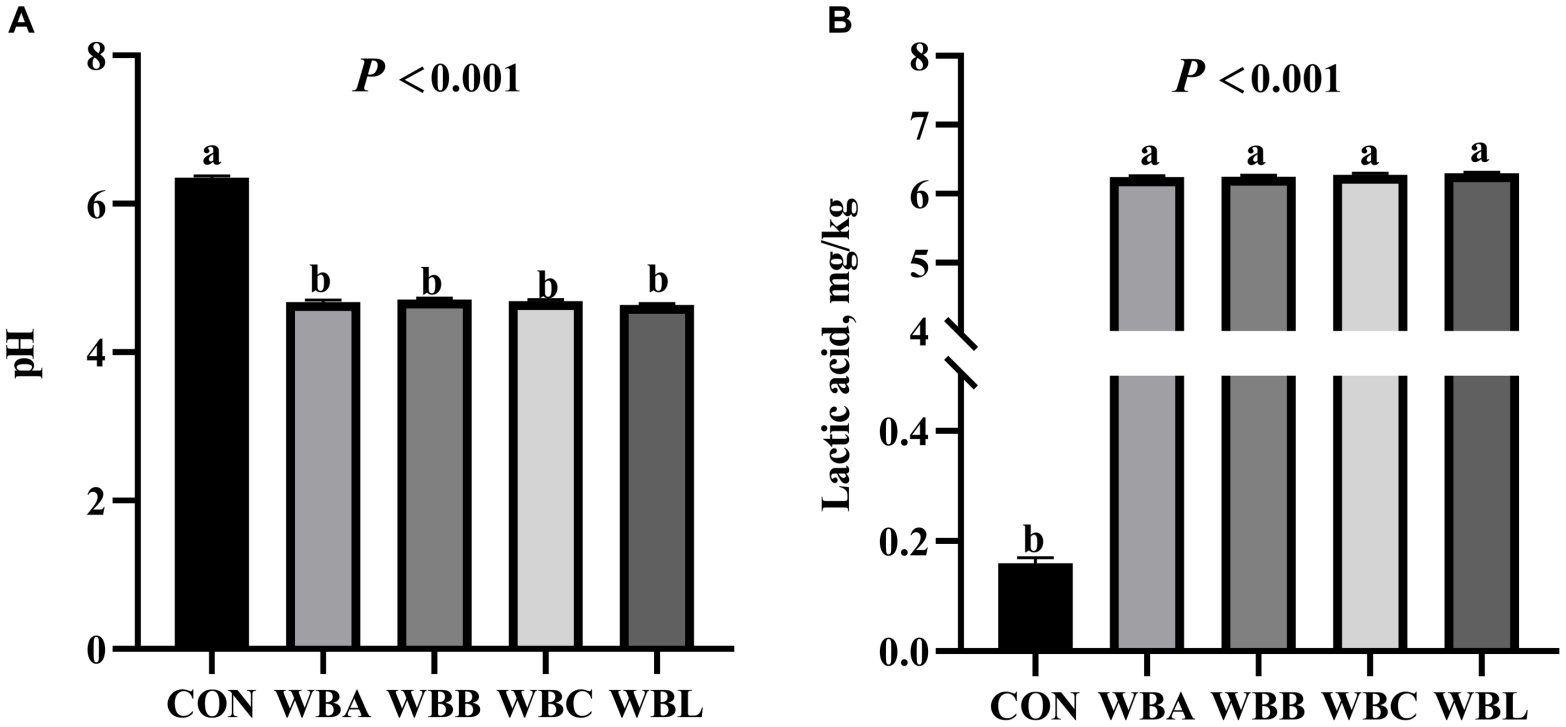
The fermentation parameters of wheat bran fermented by different strains (*n* = 8). CON is raw wheat bran, WBA, WBB, WBC, and WBL were *A. niger*, *B. licheniformis*, *C. utilis*, and *L. plantarum* fermented wheat bran, respectively. **(A)** The pH value; **(B)** The contents of lactic acid. Data are means for 8 replicates. ^a,b^Means among the experimental groups with different letters are significantly different (*p* < 0.05).

The fermentation parameters and nutrient content of wheat bran fermented by different strains are shown in Figure 1 and Table 2, respectively. Compared to CON treatment, all fermented WB treatments had lower pH values and CF content, and higher lactic acid and CP values (*p* < 0.05). Meanwhile, WBA, WBB, and WBC treatments had higher GE value compared to CON group (*p* < 0.05).

**Table 2:** Nutrient content of wheat bran fermented by different strains (DM basis, %).

Items	Treatments					SEM	*p*-value
	CON	WBA	WBB	WBC	WBL		
DM	90.75^a^	86.57^ab^	87.48^ab^	87.36^ab^	88.21^ab^	0.562	0.064
CP	16.24^b^	17.85^a^	17.89^a^	17.90^a^	17.82^a^	0.407	0.026
EE	3.93	3.89	3.74	3.76	3.84	0.032	0.069
*CF*	8.64^a^	7.65^b^	7.31^b^	7.45^b^	7.70^b^	0.419	0.015
GE	16.73^b^	16.85^a^	16.83^a^	16.84^a^	16.78^ab^	0.012	0.025

DM, dry matter; CP, crude protein; EE, ether extract; CF, crude fiber; GE, gross energy. CON was wheat bran, WBA, WBB, WBC, and WBL were wheat bran fermented by *A. niger*, *B. licheniformis*, *C. utilis*, and *L. plantarum*, respectively. Data are means for 8 replicates. SEM, Total standard error of the means. ^a,b^Means within a row with different letters are significantly different (*p* < 0.05).

### Growth performance

The effects of wheat bran fermented by different strains on the growth performance of growing pigs are shown in Table 3. Compared to the CON, WBA, WBB, and WBC treatments had higher final weight, ADG, and lower F/G (*p* < 0.05). Meanwhile, the WBB treatments had lower F/G value compared to the WBL group (*p* < 0.05).

**Table 3:** Effects of wheat bran fermented by different strains on the growth performance of growing pigs.

Items	Treatments					SEM	*p*-value
	CON	WBA	WBB	WBC	WBL		
Initial weight, kg	43.67	43.69	43.52	43.01	43.47	0.451	0.594
Final weight, kg	59.21^b^	60.91^a^	61.58^a^	60.23^a^	60.06^ab^	0.105	0.027
ADG, kg/d	0.74^b^	0.82^a^	0.86^a^	0.82^a^	0.79^ab^	0.014	0.035
ADFI, kg/d	1.89	1.97	1.98	1.98	1.93	0.023	0.213
F/G	2.54^a^	2.39^bc^	2.30^c^	2.41^bc^	2.43^ab^	0.010	0.023

ADG, average daily gain; ADFI, average daily feed intake; F/G, feed-to-gain ratio. Control was a basal diet (CON), WBA, WBB, WBC, and WBL were basal diet in which 10% wheat bran were replaced with *A. niger*, *B. licheniformis*, *C. utilis*, and *L. plantarum* fermented wheat bran (air-dry basis), respectively. Data are means for 8 replicates of 8 pigs per replicate. SEM, Total standard error of the means. ^a,b,c^Means within a row with different letters are significantly different (*p* < 0.05).

### Apparent nutrient digestibility

The effects of wheat bran fermented by different strains on the apparent nutrient digestibility of growing pigs are shown in Table 4. Compared to CON group, WBB, WBC, and WBL treatments had higher apparent digestibility of DM, while WBA and WBB treatments had higher apparent digestibility of CP (*p* < 0.05). All pigs fed fermented wheat bran diets exhibited higher apparent digestibility of GE (*p* < 0.05). There were no significant differences in the apparent digestibilities of OM and EE among all the experimental groups (*p* > 0.05).

**Table 4:** Effects of wheat bran fermented by different strains on the apparent nutrient digestibility of growing pigs %.

Items	Treatments					SEM	*p*-value
	CON	WBA	WBB	WBC	WBL		
DM	80.75^b^	82.57^ab^	85.36^a^	84.27^a^	86.14^a^	0.627	0.034
OM	83.86	84.95	85.15	85.42	85.62	0.049	0.358
CP	81.45^b^	85.19^a^	87.72^a^	84.92^ab^	84.45^ab^	0.162	0.028
EE	82.98^ab^	84.17^a^	84.25^a^	83.24^ab^	83.44^a^	0.082	0.035
GE	0.79^b^	0.81^a^	0.84^a^	0.83^a^	0.81^a^	0.125	0.02

DM, dry matter; OM, organic matter; CP, crude protein; EE, ether extract; GE, gross energy. Control was a basal diet (CON), WBA, WBB, WBC, and WBL were basal diet in which 10% wheat bran were replaced with *A. niger*, *B. licheniformis*, *C. utilis*, and *L. plantarum* fermented wheat bran (air-dry basis), respectively. Data are means for 8 replicates of 1 pig per replicate. SEM, Total standard error of the means. ^a,b^Means within a row with different letters are significantly different (*p* < 0.05).

### Serum biochemical parameters

The effects of wheat bran fermented by different strains on the serum biochemical parameters of growing pigs are shown in Table 5. Compared to CON group, WBB treatment had higher serum ALP activity (*p* < 0.05). Meanwhile, all pigs fed fermented wheat bran diets had higher values of serum ALB and TP than CON group (*p* < 0.05). There were no significant differences in serum ALT, AST, TG, and TCHO concentrations among all the experimental groups (*p* > 0.05).

**Table 5:** Effects of wheat bran fermented by different strains on the serum biochemical parameters of growing pigs.

Items	Treatments					SEM	*p*-value
	CON	WBA	WBB	WBC	WBL		
ALT, U/L	29.89	31.01	30.85	31.97	30.98	0.324	0.432
AST, U/L	33.89	32.28	31.98	33.45	34.65	0.605	0.328
ALP, U/L	156.82^b^	163.88^ab^	169.38^a^	162.10^ab^	159.20^ab^	1.520	0.035
TP, g/L	63.82^c^	64.24^ab^	65.21^ab^	66.20^a^	65.43^ab^	0.517	<0.001
ALB, g/L	32.28^b^	35.43^a^	36.07^a^	34.92^a^	36.60^a^	0.132	<0.001
TG, mmol/L	0.41	0.34	0.38	0.40	0.42	0.002	0.561
TCHO, mmol/L	2.16	2.28	2.31	2.14	2.18	0.040	0.601

ALT, alanine aminotransferase; AST, aspartate amino transferase; ALP, alkaline phosphatase; TP, total protein; ALB, albumin; TG, triglyceride; TCHO, total cholesterol. Control was a basal diet (CON), WBA, WBB, WBC, and WBL were basal diet in which 10% wheat bran were replaced with *A. niger*, *B. licheniformis*, *C. utilis*, and *L. plantarum* fermented wheat bran (air-dry basis), respectively. Data are means for 8 replicates of 1 pig per replicate. SEM, Total standard error of the means. ^a,b,c^Means within a row with different letters are significantly different (*p* < 0.05).

### Haemato-immunological blood parameters

The effects of wheat bran fermented by different strains on the haemato-immunological blood parameters of growing pigs are shown in Table 6. Compared to the CON and WBA groups, WBB and WBC treatments had higher HGB value (*p* < 0.05). The higher MCH value were observed in WBB, WBC, WBL, and WBA treatments in respective order compared to the CON group (*p* < 0.05). There were no significant differences in WBC, LYN, RBC, MCV, and MCHC among all the experimental groups (*p* > 0.05).

**Table 6:** Effects of wheat bran fermented by different strains on the haemato-immunological parameters of growing pigs.

Items	Treatments					SEM	*p*-value
	CON	WBA	WBB	WBC	WBL		
WBC, 10^9^/L	20.56	21.49	21.53	22.80	20.93	1.024	0.961
LYN, %	43.86	44.38	44.35	44.20	44.61	1.273	0.528
RBC, 10^12^/L	6.65	6.41	6.73	6.69	6.45	0.009	0.856
HGB, g/L	119.87^b^	120.20^b^	138.59^a^	140.20^a^	125.64^ab^	7.694	0.024
MCV, fL	65.01	66.06	65.38	67.68	65.87	1.438	0.597
MCH, pg	18.67^c^	19.60^bc^	21.87^a^	20.98^a^	20.58^ab^	1.357	0.015
MCHC, g/L	300.07	298.35	297.28	295.92	301.58	1.286	0.489

WBC, white blood cell count; LYN, lymphocyte ratio; RBC, red blood cell count; HGB, hemoglobin; MCV, mean corpuscular volume; MCH, mean corpuscular hemoglobin; MCHC, mean corpuscular hemoglobin concentration. Control was a basal diet (CON), WBA, WBB, WBC, and WBL were basal diet in which 10% wheat bran were replaced with *A. niger*, *B. licheniformis*, *C. utilis*, and *L. plantarum* fermented wheat bran (air-dry basis), respectively. Data are means for 8 replicates of 1 pig per replicate. SEM, Total standard error of the means. ^a,b,c^Means within a row with different letters are significantly different (*p* < 0.05).

### Antioxidant index

The effects of wheat bran fermented by different strains on antioxidant capacity of growing pigs are shown in Table 7. Compared to CON, all pigs fed fermented wheat bran diets had higher serum SOD activity and lower MDA concentration (*p* < 0.05).

**Table 7:** Effects of wheat bran fermented by different strains on antioxidant capacity in serum of growing pigs.

Items	Treatments					SEM	*p*-value
	CON	WBA	WBB	WBC	WBL		
SOD, U/mL	59.46^c^	63.41^ab^	65.62^a^	64.85^a^	62.78^ab^	1.864	<0.001
MDA, nmol/mL	2.47^a^	2.25^b^	2.17^b^	2.08^b^	2.34^b^	0.031	<0.001

SOD, superoxide dismutase; MDA, malondialdehyde. Control was a basal diet (CON), WBA, WBB, WBC, and WBL were basal diet in which 10% wheat bran were replaced with *A. niger*, *B. licheniformis*, *C. utilis*, and *L. plantarum* fermented wheat bran (air-dry basis), respectively. Data are means for 8 replicates of 1 pig per replicate. SEM, Total standard error of the means. ^a,b,c^ Means within a row with different letters are significantly different (*p* < 0.05).

### Fecal microbiota

The effects of wheat bran fermented by different strains on fecal microbiota of growing pigs are shown in Table 8. Compared to the CON group, pigs fed *B. licheniformis*, *C. utilis*, and *L. plantarum* fermented wheat bran diets had lower fecal *Escherichia coli* count (*p* < 0.05); pigs fed fermented wheat bran diets exhibited higher fecal *Lactobacillus* counts (*p* < 0.05).

**Table 8:** Effects of wheat bran fermented by different strains on fecal microbiota of growing pigs (log^10^CFU/g).

Items	Treatments					SEM	*p*-value
	CON	WBA	WBB	WBC	WBL		
*Escherichia coli*	5.85^a^	5.76^ab^	5.68^b^	5.60^b^	5.54^bc^	0.072	0.024
*Lactobacillus*	8.30^b^	8.64^a^	8.87^a^	8.71^a^	8.86^a^	0.069	0.034

Control was a basal diet (CON), WBA, WBB, WBC, and WBL were basal diet in which 10% wheat bran were replaced with *A. niger*, *B. licheniformis*, *C. utilis*, and *L. plantarum* fermented wheat bran (air-dry basis), respectively. Data are means for 8 replicates of 1 pig per replicate. SEM, Total standard error of the means. ^a,b,c^Means within a row with different letters are significantly different (*p* < 0.05).

## Discussion

Microbial fermentation is considered a highly efficient and cost-effective processing technique for improving the nutritional quality of feed (25). The pH is a crucial reference used to estimate the quality of fermented feed, and a lower pH is advantageous for the nutrient digestion and suppression of harmful bacteria growth (26). Our results showed significant lower pH values of fermented wheat brans, which might be attributed to the increase in lactic acid (27). During the fermentation, the synthesis of microbial protein and the synergistic effect of enzymes secreted by strains may lead to an increase in CP content and a decrease in CF content (28). Consistent with previous research, our data also indicated that the CP values of wheat brans after fermentation were increased, while the CF values were decreased. Additionally, the GE values of wheat bran fermented by *A. niger*, *B. licheniformis*, and *C. utilis* were significantly higher than that of unfermented wheat bran, which was similar to Liu et al. (7).

Under the action of microorganisms, complex macromolecular compounds in feed are degraded into small molecular substances, which are effectively absorbed and utilized by animals (29). Vast majority of studies have reported the positive effects of fermented feed on growth performance. For instance, Dei et al. (20) found that feed fermented by *A. niger* increased feed intake and decreased F/G of broilers. Zhao et al. (30) also proved that fermented Ginkgo-leaves by *C. utilis* and *A. niger* improved the feed conversation ratio of laying hens. Similarly, our data showed that pigs fed with WBA, WBB, and WBC diets exhibited significant increases in the final weight and ADG values, and decreases in the F/G ratio. The mechanism by which fermented feed enhanced animal growth performance of above studies might be related to its promotion of intestinal development (31). However, pigs fed with wheat bran fermented by *L. plantarum* did not exhibit significant improvement in growth performance of pigs. Consistently, Le et al. (32) also did not observe significant difference of weaned pigs fed with wheat fermented by *Lactobacillus*.

The nutrient digestibility is an important indicator associated with growth performance. In the present study, the fermented wheat bran using different strains improved the digestibility of nutrient (CP, DM, and GE) to varying degrees, which was consistent with results in previous studies related to fermented soybean meal (33, 34). It was reported that the complex macromolecular compounds of wheat bran could be degraded into small molecular nutrients after fermentation (35), and fermented wheat bran supplementation could increase the digestive enzymatic activity in the intestine. Feng et al. (36) indicated that wheat bran fermented by *Bacillus cereus* increased the activity of amylase in the duodenum of broilers. Similar results were obtained from Deng et al. (37), who demonstrated that the *Bacillus subtilis* increased the activities of amylase and lipase in the ileum of piglets. It is worth emphasizing that Cruz et al. (38) demonstrated that dietary supplementation with *C. utilis* promoted the gene expression related to nutrient transporters in jejunum of broilers. Nevertheless, intestinal morphology analysis is necessary in the further study to determine the crucial mechanism of fermented wheat bran on nutrient digestibility.

The haemato-parameters are of great significance in evaluating physical health status and predicting disease occurrence (39). Our data showed the significant increase in HGB of the WBB and WBC diets, as well as MCH of the WBB, WBC, and WBL diets. These results suggested an improvement in the body’s iron absorption, which might be associated with a reduction in anti-nutritional factors (40). Dietary fiber, due to its high phytate content, has been reported to reduce the non-heme iron absorption through chelation (41). Therefore, the improvement of HGB and MCH in pigs fed fermented wheat bran might be attributed to the degradation of CF in wheat bran after fermentation.

Serum biochemical parameters reflect the nutritional metabolism and comprehensive functions of body organs, indirectly predicting the occurrence of diseases in pigs (42, 43). The ALP not only benefits the formation of hard tissue, especially bones, but also has anti-inflammatory functions (44). We found that the wheat bran fermented by *B. licheniformis* significantly increased serum ALP activity of pigs, which was consistent with previous studies in piglets (45) and tilapia fish (46). This might be due to the presences of abundant enzyme activities (phytase and enzymes that hydrolyze non starch polysaccharide components) in fermented feed, leading to an increase in mineral intake during the ossification process (47). Serum TP and ALB levels are related to body protein metabolism (48). Higher TP levels benefit to promote tissue development, while ALB is responsible for scavenging free radicals and transporting nutrients (49). In the present study, pigs fed the fermented wheat bran sources had higher serum TP and ALB concentration, indicating an improvement in body nutritional metabolism. The changes in serum TP and ALB concentration might be linked to the alteration of quantity and quality of proteins intake after wheat bran fermentation (50). Zhao et al. (35) reported that the high molecular weight proteins were degraded into low molecular weight peptides in wheat bran fermented by *yeast*.

MDA is an advanced oxidation product and a recognized biomarker of oxidative stress, whose presence have a negative impact on growth performance of pigs (51, 52). Serum SOD is a well-known antioxidant enzyme responsible for scavenging superoxide anion radicals and converting them into hydrogen peroxide and molecular oxygen (53, 54). In the present study, pigs fed with fermented wheat bran diets showed decreased MDA level and increased SOD activity in the serum, indicating an improvement in antioxidant capacity of the body. Zhao et al. (35) reported that yeast and *Lactobacillus bulgaricus* fermentation increased the total phenols contents in wheat bran, which contributed to the improvement of antioxidant capacity of the body. Additionally, Kumari et al. (55) found that *B. licheniformis* fermentation increased antioxidant activity of soybean meal hydrolysates. In summary, our findings suggested the fermented wheat bran had potential to alter the blood profiles of growing pigs, and the mechanism behind these associations needs further research.

The fecal microbiota plays a pivotal role in safeguarding the hosts against disease resulting from bacterial colonization, promoting nutrient absorption, and maintaining intestinal morphology (56, 57). The *E. coli* is a harmful pathogen known to cause poor growth performance, diarrhea and mortality in pigs (58). On the other hand, it has been reported that *Lactobacillus casei* has the ability to regulate intestinal immune function, maintain microbial balance and reduce inflammatory reactions (59). Multiple studies have demonstrated that fermented feed has the potential to decrease the abundance of *Enterobacteriaceae* and increase the abundance of *Lactobacilli* in the gastrointestinal tract of animals (60, 61). Consistent with previous studies, our data showed a significant decrease in fecal *E. coli* counts in pigs fed the WBB, WBC, and WBL diets, while a significant increase in fecal *Lactobacillus* counts in pigs fed the fermented wheat bran diets, which might associate with the increased lactic acid content in fermented wheat bran in our study. The presences of numerous organic acids in fermented feed not only establish an optimal environment for beneficial bacteria to adhere (4), but also compete against pathogenic bacteria by freely traversing membrane, resulting in enzyme breakdown and proton movement (62, 63). In summary, our findings indicated the beneficial effects of fermented wheat bran on fecal microbial composition of growing pigs.

## Conclusion

In conclusion, our findings suggested that replacing 10% wheat bran with *B. licheniformis* fermented wheat bran could improve the growth performance, nutrient digestibility, serum antioxidant capacity, and gut microbiota composition in growing pigs. This study provides new ideas for the application of wheat bran in pig production.

## Data availability statement

The original contributions presented in the study are included in the article/Supplementary material, further inquiries can be directed to the corresponding authors.

## Ethics statement

The animal study was approved by Animal Care and Use Committee of Shandong Agricultural University (Approval Number: SDAUA-2017-0318). The study was conducted in accordance with the local legislation and institutional requirements.

## Author contributions

HL: Data curation, Formal analysis, Methodology, Project administration, Resources, Software, Writing – original draft. XR: Methodology, Writing – review & editing, Data curation, Formal analysis, Project administration, Resources. YL: Methodology, Validation, Writing – review & editing. QC: Writing – review & editing. LiY: Formal analysis, Methodology, Validation, Writing – review & editing. SJ: Conceptualization, Investigation, Methodology, Software, Validation, Writing – review & editing. JF: Data curation, Project administration, Resources, Writing – review & editing. JG: Conceptualization, Funding acquisition, Investigation, Supervision, Writing – review & editing. LeY: Writing – review & editing. JL: Conceptualization, Investigation, Visualization, Writing – review & editing. WY: Conceptualization, Funding acquisition, Investigation, Supervision, Visualization, Writing – review & editing.
