# Supplementary material for: Effects of different wheat bran fermentation sources on growth performance, nutrient digestibility, serum antioxidant capacity and fecal microbiota in growing pigs
Source: Front Vet Sci. 2023 Nov 16;10:1289010. doi: 10.3389/fvets.2023.1289010 (PMC10687151; doi:10.3389/fvets.2023.1289010)
Supplement: Supplementary file 1 [file Data_Sheet_1.PDF]

Supplementary Material

Effects of different wheat bran fermentation sources on growth performance, nutrient digestibility, serum antioxidant capacity and fecal microbiota in growing pigs

Heng Liu [1], Xiaojie Ren [2], Yang Li [1], Qingjie Cao [3], Lijie Yang [1], Shuzhen Jiang [1], Jiawei Fu [1], Jie Gao [4], Lei Yan [5], Junxun Li [2*], Weiren Yang [1,*]

[*] **Correspondence:** Weiren Yang, E-mail: wryang@sdau.edu.cn; Junxun Li, E-mail: 892686923@qq.com.

Supplementary Table S1. The cultivation methods of strains.

Strains	Medium	Composition	Tempe rature	Ti me	Shaker Speed
Aspergillus niger	Yeast Extract Peptone Dextrose Medium (YPD)	Yeast extract 10g, Peptone 10g, Dextrose 20g, H_2O 1000mL	30°C	72 h	—
Bacillus licheniformis	0.2% dextrose medium	Dextrose 2g, Peptone 10g, Yeast extract 5g, NaCl 5g, H_2O 1000 mL, pH 7.0	37°C	24 h	—
Candida utilis	Potato Dextrose Agar medium (PDA)	200 g potato was supplemented with water, boiled for 30 mins and collected the juice to a volume of 1000 mL, 20 g Dextrose, Natural pH	32°C	24 h	—

Lactobacillus plantarum	Mann-RogosaSharpe(MRS)	$C_6H_5O_7(NH_4)_3$ 2g, $C_2H_3NaO_2 \cdot 3H_2O$ 5g, K_2HPO_4 5g, $MgSO_4$ 0.5g, $MnSO_4$ 0.2g, Dextrose 20g, Peptone 10g, Beef extract 10g, Yeast extract 5g, tween 80 1g, H_2O 1000mL, Natural pH	37°C	24 h	120 r/min

Supplementary Table S2. The fermentation methods of wheat bran.

Treatments	Inoculation proportion	Temperature	Time	Aerobic/Anaerobic conditions	Reference
WBA	5% *Aspergillus niger* (1×10^9 CFU/mL)	30 ℃	32 h	Aerobic fermentation	(18-21)
WBB	5% *Bacillus licheniformis* (2×10^{10} CFU/mL)	37 ℃	48 h	Aerobic fermentation	
WBC	5% *Candida utilis* (1× 10^9 CFU/mL)	32 ℃	28 h	Anaerobic fermentation	
WBL	5% *Lactobacillus plantarum* (1×10^{10} CFU/mL)	37 ℃	48 h	Anaerobic fermentation	
